# Quantifying the relative importance of experimental data points in parameter estimation

**DOI:** 10.1186/s12918-018-0622-6

**Published:** 2018-11-22

**Authors:** Jenny E. Jeong, Peng Qiu

**Affiliations:** 1 Electrical and Computer Engineering, Georgia Institute of Technology, Atlanta, 30332 GA USA; 2 Biomedical Engineering, Georgia Institute of Technology and Emory University, Atlanta, 30332 GA USA

**Keywords:** Weighted least squares, Parameter estimation, Ordinary differential equation

## Abstract

**Background:**

Ordinary differential equations (ODEs) are often used to understand biological processes. Since ODE-based models usually contain many unknown parameters, parameter estimation is an important step toward deeper understanding of the process. Parameter estimation is often formulated as a least squares optimization problem, where all experimental data points are considered as equally important. However, this equal-weight formulation ignores the possibility of existence of relative importance among different data points, and may lead to misleading parameter estimation results. Therefore, we propose to introduce weights to account for the relative importance of different data points when formulating the least squares optimization problem. Each weight is defined by the uncertainty of one data point given the other data points. If one data point can be accurately inferred given the other data, the uncertainty of this data point is low and the importance of this data point is low. Whereas, if inferring one data point from the other data is almost impossible, it contains a huge uncertainty and carries more information for estimating parameters.

**Results:**

G1/S transition model with 6 parameters and 12 parameters, and MAPK module with 14 parameters were used to test the weighted formulation. In each case, evenly spaced experimental data points were used. Weights calculated in these models showed similar patterns: high weights for data points in dynamic regions and low weights for data points in flat regions. We developed a sampling algorithm to evaluate the weighted formulation, and demonstrated that the weighted formulation reduced the redundancy in the data. For G1/S transition model with 12 parameters, we examined unevenly spaced experimental data points, strategically sampled to have more measurement points where the weights were relatively high, and fewer measurement points where the weights were relatively low. This analysis showed that the proposed weights can be used for designing measurement time points.

**Conclusions:**

Giving a different weight to each data point according to its relative importance compared to other data points is an effective method for improving robustness of parameter estimation by reducing the redundancy in the experimental data.

**Electronic supplementary material:**

The online version of this article (10.1186/s12918-018-0622-6) contains supplementary material, which is available to authorized users.

## Background

To understand and study the properties of biological systems, mathematical modeling has been a powerful tool [1, 2]. For example, ordinary differential equations (ODEs), Bayesian networks, Boolean networks, Petri nets, and many other mathematical forms [3–6] have been used to describe biological processes. Since the ODE-based modeling is useful in representing the changes of interacting components of dynamical systems over time [7], it is often used to model gene and protein networks in systems biology.

ODE-based systems biology models are often constructed by encoding prior knowledge of interactions among individual components in the systems. Such ODE models typically contain many unknown model parameters, such as reaction rates, binding affinities and Hill coefficients [8, 9], as well as many dynamic variables nonlinearly interacting with each other. The unknown model parameters can be estimated based on the experimentally observed data. Given an ODE model and experimental data, parameter estimation can be formulated by minimizing the least squares cost function Eq. (1), which we consider as equal-weight cost function,

1 $$ Cost=\frac{1}{2}\ \sum_{i=1}^{n}({obs}_{i} - {pred}_{i}(\theta))^{2}   $$

where *n* represents the total number of experimentally observed data points, *obs*_*i*_ represents the value of the *i*^*th*^ observed data point, *pred*_*i*_ represents the model prediction of the *i*^*th*^ data point which is a function of the parameters *θ*. This equal-weight cost function measures the differences between experimental data and model predictions obtained from the estimated parameters. By minimizing the cost function, we can identify optimal parameters that enable the model to produce predictions of simulations that resemble the experimental data. Some studies included weights in the least squares Eq. (2) to account for measurement noise [10],

2 $$ Cost=\frac{1}{2}\ \sum_{i=1}^{n} w_{i} ({obs}_{i} - {pred}_{i}(\theta))^{2}   $$

where *w*_*i*_ is inversely related to the expected measurement noise associated to the *i*^*th*^ observed data point. This formulation assigns lower weights to data points with larger expected measurement noise, so that the cost function focuses on the more accurately measured data points. Although Eq. 2 assigns different weights to each data point, it still treats all data points intrinsically equally important and the weights just reflects our ability of measuring different data points.

In systems biology, ODE models are usually highly complex, whereas the amounts of available experimental data are almost always limited. This imbalance between the complexity of the models and the insufficient experimental data makes parameter estimation a very challenging problem. It is possible that drastically different parameter settings can fit the experimental data equally well, which is a manifestation of an information gap between the model complexity and the available experimental data [11].

One intuitive approach to address the parameter estimation challenge in systems biology is to obtain more data. Cubic spline interpolation was used to generate new data by increasing the time resolution [12]. By interpolating time points in–between already observed experimental data points, the optimization cost function can be defined on densely interpolated time points. New data can also be generated via experimental design, which aims to identify the most informative new experiments that efficiently increase the information content in the data [13–16]. Examples include minimizing the uncertainty of parameters using sensitivity analysis [14, 17], maximizing mutual information between parameter and data from new experiments [15, 18], and maximizing the variance of new experiments with respect to the Bayesian posterior distribution of the parameters [16, 19–21]. A common concept in these studies is that the new data obtained by various potential new experiments are expected to provide different amounts of information. Following the same logic, different data points observed in the same experiment probably also provide different amount of information, which motivated us to treat each data point differently by considering their relative importance, instead of treating them equally.

**Fig. 1:**
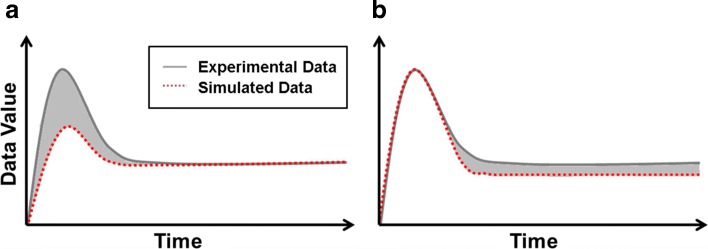
Illustrative example showing limitations of the equal-weight cost function. The solid curve represents the experimental data, and the dotted curve represents model prediction based on a parameter estimate. **a** A poor fit that does not capture the dynamic behavior but fits well to the flat region. **b** A better fit that captures the dynamic region well but is slightly off in the flat region. The shaded area represents the value of the cost function if all data points are considered as equally important. Depending on the length of the flat region, the two shaded areas (costs) can be equivalent. Therefore, the equal-weight cost function is not able to distinguish these two parameter estimates

Although typical parameter estimation studies treat all data points as equally important, it can give rise to misleading results as illustrated in Fig. 1. In this illustrative example, we ignore the measurement error and consider the equal-weight cost function Eq. (1). In Fig. 1a and b, the solid curves represent the same experimentally observed data, and the dotted curves represent model predictions based on different parameter settings. In Fig. 1a, the dotted curve fits the steady state accurately, whereas the dynamical region of the behavior is not well captured. If we measure the quality of the fit by the equal-weight cost function, the value of the cost is represented by the shaded area in-between the two curves. Figure 1b shows the model prediction based on a different parameter setting. In comparison with Fig. 1a, the parameter setting in Fig. 1b produces a better fit because it captures the dynamical behavior accurately, although it is slightly off at the steady state. In cases where the steady state region lasts for a long period of time, the cost value of the second parameter (Fig. 1b) can be the same as the cost value in (Fig. 1a) or even larger. This example shows that if all data points are considered equally important, the cost function defined by Eq. (1) is not able to distinguish a poor fit from a good fit. Instead of the equal-weight formulation, if the weights are strategically distributed to give higher emphasis to data points in the dynamic region and lower emphasis to data points in the steady state region, the weighted cost function will be able to favor the parameter setting in Fig. 1b over that in Fig. 1a, regardless of the length of the steady state region.

In order to consider the relative importance of data points when formulating the parameter estimation cost function, we propose to define a weight for each data point by the amount of unique information it provides. More specifically, we define the weight of a data point by the uncertainties of the data point given all other data points. This uncertainty quantifies how well we can infer one data point given the other data points. For instance, if one data point can be accurately predicted given the other data points, its uncertainty is low, and it carries a very small amount of new information beyond what other data points provide. On the other hand, if one data point cannot be accurately inferred given the other data points, this data point has high uncertainty and carries unique information beyond other data points. Defining weights by the uncertainty leads to a weighted cost function, where each data point is weighted by the amount of unique information it provides. To examine this weighted cost function, the G1/S transition model and the MAPK module were used. In addition, a sampling algorithm was developed to evaluate and compare the equal-weight formulation and the proposed uncertainty-weighted formulation.

## Methods

### Parameter uncertainty given experimental data

Before introducing weights defined by the uncertainty of a data point given the other data points, we first discuss the uncertainty of parameters given data points. Assume we have an optimal parameter setting (*θ*^⋆^) which minimizes the weighted cost function Eq. (2). In a small neighborhood of *θ*^⋆^, there exists a region of parameter settings that, although not optimal, are nevertheless consistent with the data within experimental noise. These near optimal parameter settings form the confidence interval for the estimated optimal parameter, and the corresponding variation in these near optimal parameters is the uncertainty of parameters, which can be estimated by a second-order Taylor expansion of the cost function Eq. (2) at the optimal parameter setting.

3 $$\begin{array}{@{}rcl@{}} C(\theta) &\approx& C(\theta^{\star})+\frac{1}{2}\sum_{a=1}^{m}\sum_{b=1}^{m} \frac{\partial^{2} C}{\partial \theta_{a} \partial \theta_{b}}(\theta_{a} - \theta_{a}^{\star})(\theta_{b} - \theta_{b}^{\star}) \\ &=& C(\theta^{\star})+\frac{1}{2}(\theta - \theta^{\star})^{T}H(\theta - \theta^{\star}) \end{array} $$

In Eq. (3), *m* represents the number of parameters. The first-order term does not appear in the Taylor expansion because the gradient of the cost function at the optimal parameter (*θ*^⋆^) is 0. The second-order derivatives evaluated at the optimal parameter (*θ*^⋆^) is the Hessian (*H*) at the optimal parameter. The eigenvalues and eigenvectors of the Hessian matrix reflect the confidence interval of the near optimal parameters. For example, if the Hessian has a small eigenvalue, moving the optimal parameter along the corresponding eigenvector direction does not significantly increase the cost value, leading to a huge confidence interval and a large uncertainty of the optimal parameter. On the other hand, if the eigenvalues of the Hessian are all large, moving the optimal parameter in any eigendirection will lead to large increase in the cost value, indicating a very small confidence interval and small uncertainty of optimal parameter.

The Hessian matrix can be approximated by the Fisher Information Matrix (FIM), vis the simplifications as follows Eq. (4),

4 $$ {{\begin{aligned} H_{a,b} &= \frac{\partial^{2} C}{\partial \theta_{a} \partial \theta_{b}} \left|{\!~\!}_{\theta^{\star}}\right. \\ & \left.=\sum_{i=1}^{n}\frac{\partial}{\partial \theta_{b}} \left(({obs}_{i} - {pred}_{i}(\theta))(-w_{i}) \frac{\partial {pred}_{i}(\theta)}{\partial \theta_{a}} \right) \right|_{\theta^{\star}} \\ &=\sum_{i=1}^{n} \left(w_{i}\frac{\partial {pred}_{i}}{\partial \theta_{a}}\frac{\partial {pred}_{i}}{\partial \theta_{b}} - w_{i}({obs}_{i}-{pred}_{i}(\theta))\frac{\partial^{2} {pred}_{i}}{\partial \theta_{a} \partial \theta_{b}} \right) \left|{\!~\!}_{\theta^{\star}}\right. \\ & \left. \approx \sum_{i=1}^{n}w_{i}\frac{\partial {pred}_{i}}{\partial \theta_{a}}\frac{\partial {pred}_{i}}{\partial \theta_{b}} \right|_{\theta^{\star}} \end{aligned}}}  $$

In Eq. (4), *n* represents the total number of data points. In the final line of Eq. (4), the approximation is based on the assumption that the fit error is very small at the optimal parameter. This approximation of Hessian is the Fisher Information matrix, and its inverse is the covariance matrix that approximates the uncertainty of the optimal parameter given the data. The parameter uncertainty can be quantified using Eq. (5),

5 $$\begin{array}{@{}rcl@{}} Uncertainty(\theta|data) = \frac{1}{m}trace\left(I^{-1}\right) \end{array} $$

where *m* is the number of parameters and *I* is the Fisher Information matrix.

Since parameters in systems biology models are often constrained to be non-negative, it is often advantageous to compute the Fisher Information matrix in the log-parameter space: *I*_*a*,*b*_ = $\sum _{i=1}^{n}w_{i}\frac {\partial {pred}_{i}}{\partial \log (\theta _{a})}\frac {\partial {pred}_{i}}{\partial \log (\theta _{b})}|_{\theta ^{\star }}$. In addition, the Fisher Information matrix can be represented by *J*^*T*^*J*, where the *J* represents the Jacobian matrix. Therefore, the eigenvalues of Fisher Information matrix are equal to the squares of the singular values of the Jacobian, and the Eq. (5) can be calculated by $\sum _{a=1}^{m}\frac {1}{{s_{a}}^{2}}$, where *s* indicates the singular values of the Jacobian.

### Data uncertainty given other data

Similar to the formulation of parameter uncertainty given data, we can express the uncertainty of estimating one set of data points (*S*_1_) given another set of data points (*S*_2_) using the Fisher Information:

6 $$ Uncertainty(data S_{1}|data S_{2}) = \frac{1}{m}trace\left(I_{S1}{I_{S2}}^{-1}\right)   $$

where $[I_{S1}]_{a,b} = \sum _{i\in S1}w_{i}\frac {\partial {pred}_{i}}{\partial \log (\theta _{a})}\frac {\partial {pred}_{i}}{\partial \log (\theta _{b})}|_{\theta ^{\star }}$, and $[I_{S2}]_{a,b} = \sum _{i\in S2}w_{i}\frac {\partial {pred}_{i}}{\partial \log (\theta _{a})}\frac {\partial {pred}_{i}}{\partial \log (\theta _{b})}|_{\theta ^{\star }}$. *θ*^⋆^ here is the best fit parameter defined by data points in *S*_2_. The matrix inverse and multiplication inside the *trace* operation in Eqn. (6) approximate the derivatives of data points in *S*_2_ with respect to data points in *S*_1_.

To quantify the importance of a data point, we propose to calculate the uncertainty of one data point *i* given all the other data points. We define the two subsets as follows: *S*_1_={*i*} and *S*_2_={1,2,...,*n*}∖*S*_1_. The Fisher Information matrices *I*_*s*1_ and *I*_*s*2_ are computed using the *i*^*th*^ row of the Jacobian and all the other (*n*−1) rows of Jacobian, respectively. The data uncertainty reflects whether one data point can be accurately predicted based on all other data points. As shown in Eq. (6), calculating the weight of a data point requires the Fisher Information matrix evaluated at the optimal parameter, which is in turn defined by optimizing the weighted least squares cost function Eq. (2) that requires the weights.

### Iterative algorithm for quantifying the weight of each data point

**Fig. 2:**
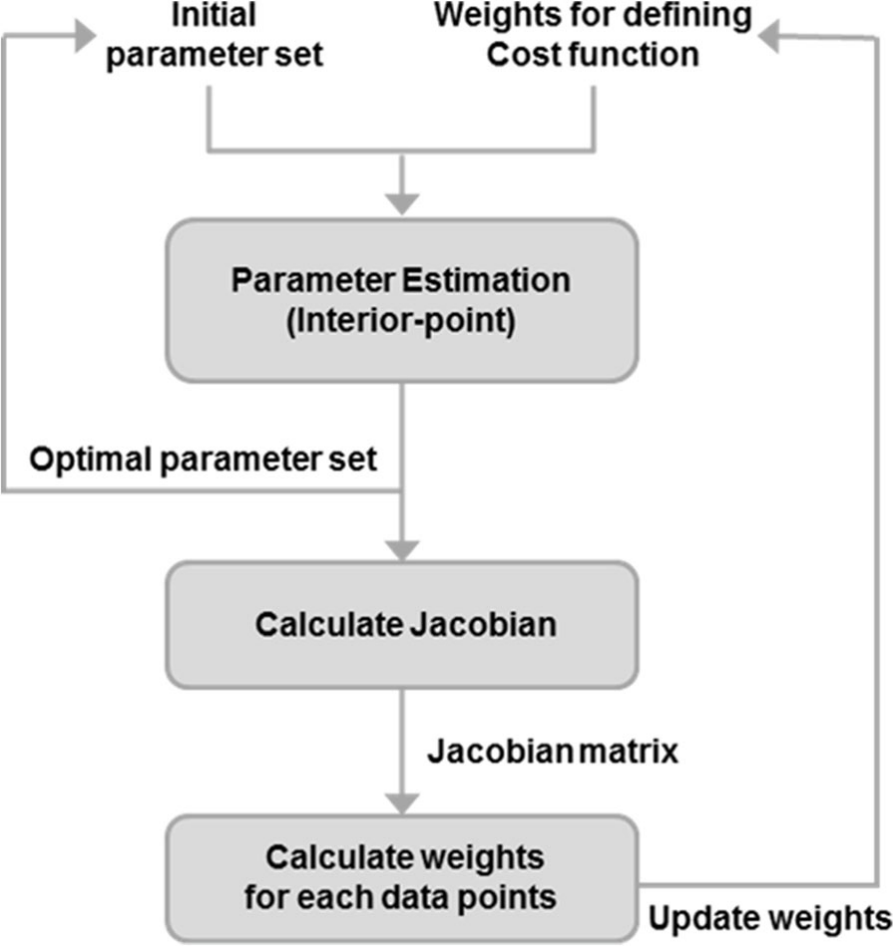
Iterative algorithm to compute uncertainty-based weights. The first iteration: parameter estimation is performed based on the equal-weight cost function using 100 random initial parameter settings obtained by Latin hypercube sampling. We pick the one with the smallest cost to calculate weights. The second and subsequent iterations: the algorithm starts with the optimized parameter from the previous iteration, and performs parameter estimation with respect to the weighted cost function using the weights from the previous iteration. The process iterates until the weights converge

To compute the weight of each data point and estimate the optimal parameter setting, an iterative algorithm is developed. Figure 2 shows a flowchart of the proposed iterative algorithm. In the first iteration, the algorithm is initialized by assigning equal weights, 1, to all data points. To optimize the parameters with respect to the initial equal-weight cost function Eq. (1), the interior-point algorithm [22] is used with randomly generated initial parameters. We evaluate the Jacobian at the estimated parameter, which is used for calculating the uncertainty associated to each data point Eq. (6). The uncertainty of each data point serves as its updated weight. We normalize the weights, so that the sum of the weights equals the total number of data points. Such normalization makes the weighted cost function and the equal-weight cost function comparable.

In the subsequent iterations, the estimated parameter and the updated weights from the previous iteration serve as the initial parameter and weights for the parameter estimation step. Therefore, the parameter estimation is performed with respect to the updated weights. After that, the weights are re-computed based on the newly estimated parameter. This process is repeated until the weights converge.

The intuition of this algorithm is that: even if the optimal parameter setting derived from the equal-weight cost function in the first iteration is incorrect, as long as it represents a decent fit to the data, the updated weights at the end of the first iteration will roughly capture the curvature and relative importance of the data points. The subsequent iterations start with the updated weights, and will gradually adjust and fine-tune the optimal parameter, as well as the weights until convergence. In practice, to ensure the first iteration obtains a decent fit, we typically perform 100 runs of parameter estimation using random initial parameters generated by the Latin hypercube sampling method. We pick the best estimated parameter among the 100 to compute the Jacobian and updated weights. The second and subsequent iterations pursue the best fit in the first iteration and fine-tune it until weights converge, which typically takes only a few iterations.

### A parameter sampling algorithm

To evaluate the benefit of the weighted cost function and compare with the equal-weight cost function, we developed a parameter sampling algorithm, similar to the Markov Chain Monte Carlo algorithm [23]. The sampling algorithm generates a collection of near optimal parameter settings. Given an acceptance threshold for near optimal, the sampling algorithm identifies the acceptable parameter region which is defined as the union of all parameter settings whose cost value is smaller than the given acceptance threshold. Although most of the acceptable parameter settings are not optimal, they generate descent model predictions which fit well to the data. Thus, this acceptable parameter region can be used to visualize the confidence interval of the estimated parameter and the model predictions.

The algorithm is as follows:

1. Define the acceptance threshold as *K* times cost value at current estimated parameter (*θ*^*curr*^).
  *Threshold*=*K*∗*cost*(*θ*^*curr*^), where *K* is the constant number.
2. Define the perturbed parameter based on the Fisher Information matrix (FIM) at the current estimated parameter.
  $\theta ^{pert} = \theta ^{curr} + \alpha \sum ^{m}_{a=1}\frac {1}{\lambda _{a}}V_{a}$, where *α* ∼ N (0,*σ*^2^), *λ* = eigenvalue of FIM, and V = eigenvector of FIM.
3. If cost value of *θ*^*pert*^ is smaller than *Threshold*, we remember the *θ*^*pert*^ as a acceptable parameter, and update to the *θ*^*curr*^. Then, we increase the *σ* two times, and re-explore parameter space with the updated *θ*^*curr*^ and *σ*.
  If cost value of *θ*^*pert*^ is bigger than *Threshold*, we reject the *θ*^*pert*^ and decrease the *σ* as a half. Then, we re-explore parameter space with the *θ*^*pert*^ and decreased *σ*.
  If the *σ* is too small, we randomly select one of the accepted parameter sets and use the selected parameter as a *θ*^*curr*^.
4. Go back to the step 2 until the total iteration number is 10^5^.

In step 1, it starts with the estimated parameter setting obtained from optimizing the weighted cost function, which serves as a seed inside the acceptable parameter region. Denote the seed as the called current parameter *θ*^*curr*^. In step 2, to explore the parameter space, we perturb the current parameter, and we evaluate the cost function at the perturbed parameter. In step 3, if the cost value is smaller than the acceptance threshold, the perturbed parameter is accepted and becomes the current parameter. On the other hand, if the cost value is larger than the threshold, the perturbed parameter is rejected and the current parameter is not changed. This sampling algorithm is performed iteratively, resulting in a collection of acceptable parameters.

In order to achieve efficient sampling and low rejection rate, we designed the direction and amplitude of the perturbation using the Fisher Information Matrix and parameter uncertainty. At each iteration of the sampling algorithm, we compute the inverse of the Fisher Information matrix at the current parameter. We apply larger perturbation along the eigendirection associated to large eigenvalues, and smaller perturbation along the eigendirection associated to small eigenvalues. Since the inverse of the Fisher Information Matrix approximates the covariance of the estimated parameter, such a choice of perturbation leads to larger perturbations along the insensitive directions that have little influence on the cost function, and smaller perturbations along the sensitive direction, enabling efficient exploration even when the covariance is highly anisotropic.

To determine the amplitude of the perturbation, we use a normal distribution, *N*(0,*σ*^2^). A large *σ* value makes the sampling algorithm explore the parameter space quickly, but is at the risk of low acceptance rate and low sampling efficiency. On the other hand, a small *σ* value has the opposite effect. In the sampling algorithm, the value of *σ* is adjusted during the process, doubled when the perturbed parameter is accepted and halved when the perturbed parameter is rejected. The purpose of adjusting *σ* is to balance between the exploration and the acceptance rate. Furthermore, if the *σ* value is too close to zero, meaning that the sampling process is stuck at a narrow corner of the acceptable parameter region, we randomly pick a previously found acceptable parameter and set is as the current parameter. This heuristic effectively resets the sampling process when it is stuck.

## Results

### G1/S transition model

**Fig. 3:**
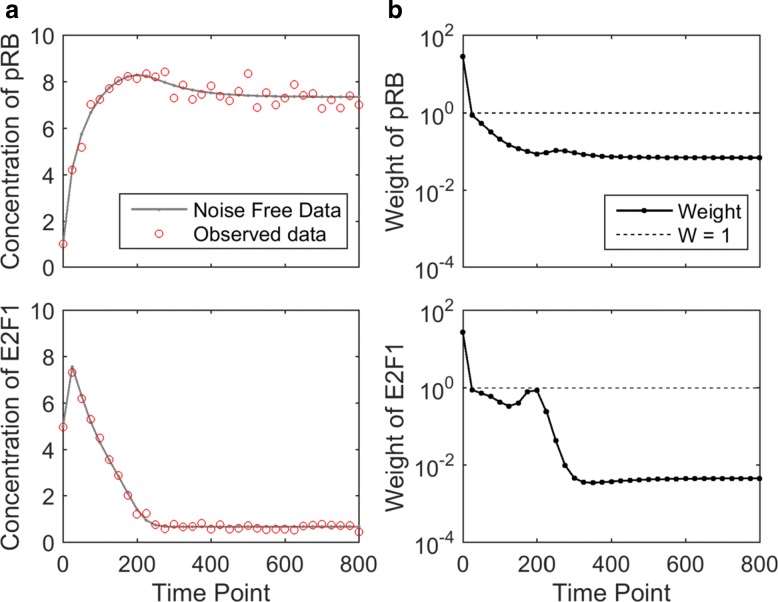
Experimental data and weights of the G1/S transition model with 6-parameters. **a** The solid curve represents the simulated noise-free data obtained from the true parameter. The circles represent simulated experimental data, which is obtained by introducing a small amount of Gaussian noise. This noisy data is used as the observed experimental data in parameter estimation. **b** Each dot represents the weight of a data point, and the dashed line corresponds to the weights in the equal-weight cost function (“1”). All curves are shown in log scale. The dynamic region receives relatively larger weights and the flat region receives relatively smaller weights

To test the proposed algorithm for computing uncertainty-based weights, the G1/S transition model was used [12, 24]. This model consists of 2 variables, pRB (Retinoblastoma protein) and E2F1 (Activator), and 10 model parameters. We also consider the initial concentrations of both variables as parameters, and therefore, the total number of model parameters is 12. The ordinary differential equations of the model are described in Eq. (7). Since pRB inhibits E2F1 activation, the concentration of E2F1 decreases as the concentration of pRB increases as shown in Fig. 3a. Afterwards, the concentrations of both variables approach steady state gradually.

7 $$ {\begin{aligned} \frac{d}{dt}[pRB]&=K_{1}\frac{[E2F1]}{K_{n1}+[E2F1]}\frac{J_{11}}{J_{11}+[pRB]}-\varphi_{pRB}[pRB]\\ \frac{d}{dt}[E2F1]&\,=\,K_{p}\,+\,K_{2}\frac{a^{2}+[E2F1]^{2}}{K_{n2}^{2}+[E2F1]^{2}}\frac{J_{12}}{J_{12}+[pRB]}\,-\,\varphi_{E2F1}[E2F1] \end{aligned}}  $$

To generate experimental data, we simulated Eq. 7 using the parameter setting in [12] as the true parameter, with observed time points evenly spaced every 25 min from 0 to 800 min. The simulated data was perturbed with a small amount of multiplicative and additive Gaussian noise [14]. Figure 3a shows the simulated experimental data. The total number of experimental data points is 66 (33 data points for each variable). Based on this experimental data, we examined two versions of the model, one with 6 unknown parameters, and the other with 12 unknown parameters. For both versions of the model, the initial concentrations of the two variables were treated as unknown parameters.

#### G1/S transition with 6 unknown parameters

To consider a simple version of the G1/S transition model, four model parameters (*K*_1_,*J*_11_,*K*_*n*2_ and *J*_12_) and the initial concentrations of both variables were treated as unknown parameters. Using the iterative algorithm described in the “Methods” section, weights of the 66 data points were calculated and visualized in Fig. 3a. The solid curve represents the weight of each data point and the horizontal dotted line represents the equal-weight formulation where every data point receives the same weight 1. As shown in Fig. 3b, the initial data point (*t*=0) of both variables received the highest weights among all data points. This is because the initial data points directly reflect values of two unknown parameters, which are the initial concentrations. Among other data points, those in dynamically changing regions (from ∼25 to ∼250 mins) received relatively larger weights compared to those in flat regions (from ∼250 to ∼800 mins).

**Fig. 4:**
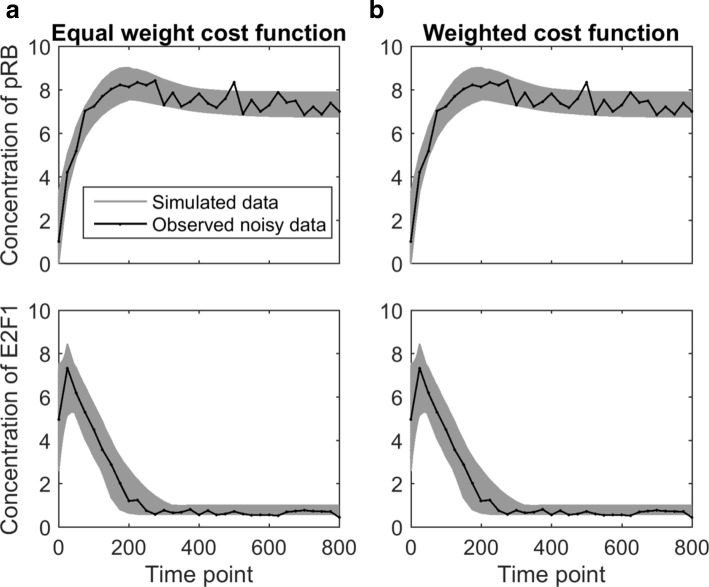
Sampling algorithm for evaluating G1/S transition with 6-parameters. The black curve indicates the observed experimental data. The gray curves represent the model predictions based on the acceptable parameter settings, collectively forming a gray belt. **a** Sampling results with respect to the equal-weight cost function. The E2F1 belt from the equal-weight cost function shows imbalance between the thick width in the dynamic region and the thin width in the flat region. **b** Sampling results obtained from the weighted cost function. The belt width of E2F1 is much thinner in the dynamic region because the weighted cost function gives higher weights to the data points in the dynamic region. At the early time points where pRB exhibits bigger change, the pRB belt for the weighted cost function is thinner than that for the equal-weight cost function

To compare the equal-weight cost function and the weighted cost function, the sampling algorithm introduced in the “Methods” section was applied to visualize the collection of acceptable parameter settings. For each cost function, the interior-point algorithm [22] was used to estimate the underlying parameter. The estimated parameter setting was then used as the seed for the sampling algorithm to generate collections of acceptable parameters. We defined the threshold for acceptable to be three times the cost value of the estimated parameter setting. Finally, we simulated the model using the acceptable parameters. Figure 4 visualizes the model predictions generated from the acceptable parameters for both cost functions. The gray belt represents the range of acceptable model predictions and the black curve is the experimental data (Fig. 3a).

Figure 4a represents the model predictions of the collection of acceptable parameters derived by the sampling algorithm with the equal-weight cost function. Figure 4b corresponds to the weighted cost function. From this figure, we can see that the belts corresponding to the second variable (E2F1) are quite different between the equal-weight and weighted cost functions. The equal-weight cost function resulted in a belt that is relatively thick in the dynamic region, and relatively thin in the flat region. This is because the large number of data points in the flat region essentially carries the same information, the steady state, forcing the parameter estimation algorithm to focus on finding the steady state accurately, even at the expense of errors in the dynamic region. This unbalanced belt width illustrates the limitation of the equal-weight cost function described in Fig. 1. The belt width of the first variable (pRB) is more balanced, because the flat region of the first variable is shorter than the second variable, and the data points for pRB are noisier than those for E2F1. For the weighted cost function, the belt is much thinner in the dynamic region. This is because data points in the dynamic region received larger weights while data points in the flat region received smaller weights. By redistributing the weights according to the relative importance of each data point, we can derived the weighted cost function, giving larger weights to the data points in dynamically changing regions and lower weights to flat regions.

**Fig. 5:**
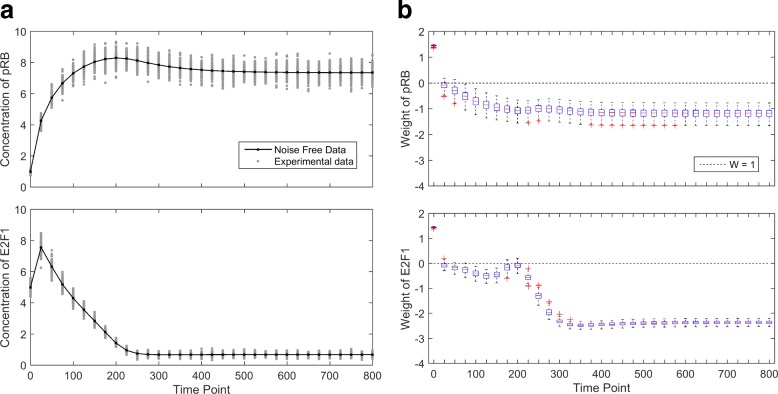
G1/S transition with 6-parameters: robustness of the uncertainty-based weights. **a** The black curve represents the noise-free data and the gray dots represent 100 simulated noisy datasets for sensitivity analysis. **b** The dotted line represents the weight of equal-weight cost function (“0” on log scale), and each box represents the weights for one data point, computed from the 100 noisy experimental datasets. The small range of each box indicates the robustness of the uncertainty-based weights

To test the sensitivity of the algorithm for computing the weights, we generated 100 experimental datasets by randomly perturbing the noise-free simulation in Fig. 3a. The variation among the 100 datasets is shown in Fig. 5a. Using the iterative algorithm, weights are computed based on each experimental dataset. The variation among the 100 sets of weights is shown in Fig. 5b. The first measurement time point for both variables consistently receive large weights across the 100 datasets, very robust to the noise. For other measurement time points, we can observe the same pattern as in Fig. 3b, where the dynamically change regions consistently receive higher weights than flat regions.

#### G1/S transition with 12 unknown parameters

To consider a more challenging situation, we assumed that all 12 parameters are unknown. Now we have to estimate 12 unknown model parameters with the same experimental data (Fig. 3a), and perform the iterative algorithm to calculate the weights. The resulting weights are shown in Additional file 1: Figure S1, which is qualitatively similar as before (larger in dynamic regions and lower in flat regions), but not exactly the same. This shows that the uncertainty-based weights do not just simply encode the curvature of the given data. They are also influenced by the mathematical structure of the ODE-model and which parameters need to be estimated.

Additional file 1: Figure S2 illustrates the model predictions of acceptable parameters for both cost functions, using the sampling algorithm. The comparison is qualitatively the same as the previous 6-parameter example. The equal-weight cost function (Additional file 1: Figure S2A) led to highly unbalanced belt width because of the large number of data points in the steady state region, whereas the weighted cost function (Additional file 1: Figure S2B) led to relatively balanced belt width by assigning larger weights to data points in dynamic regions and lower weights to the data points in flat regions.

To examine the sensitivity of the weights in the 12-parameter model, we used the same 100 experimental datasets introduced in the previous example (Fig. 5a), and calculated weights based on the 100 datasets. The variation of the weights is shown in Additional file 1: Figure S3. Comparing Fig. 5b and Additional file 1: Figure S3, we can see that weights of the data points in this 12-parameter model are not as robust as the weights in the previous 6-parameter model. This is caused by the higher complexity of the 12-parameter model, making the parameter estimation component of the iterative algorithm to overfit to the noise and subsequently lead to different weights.

#### Unevenly spaced time points

**Fig. 6:**
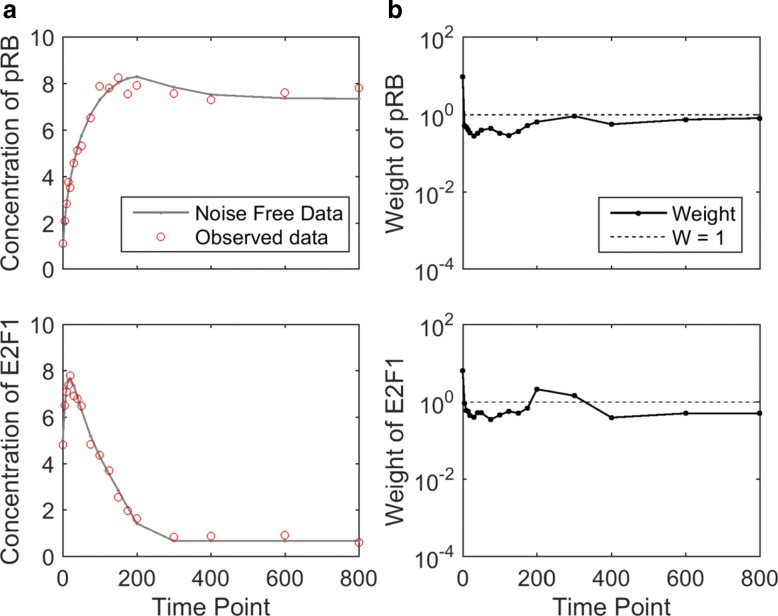
Expreimental data and weights of the G1/S transition model with unevenly spaced time points. **a** The measurement time points are selected strategically based on the weights of the evenly spaced case. The gray dot and solid line represent the simulated noise-free data obtained from the true parameter, and the circles represent the noisy experimental data. **b** The black dots and solid curve represent the weights of the data points, and the dashed line represents the equal-weight cost function. The weights are very close to the dashed line except the first time point, meaning that the chosen time points are roughly equally important based on their uncertainty quantifications

In the 6-parameter and 12-parameter models above, the experimental data points are evenly spaced along the time axis. As shown in Fig. 3b and Additional file 1: Figure S1, data point in different time periods receive different weights, and the magnitudes of weights decrease as time increases, indicating that the data points located at later time points may be redundant. Therefore, to reduce the effect of these redundant data points, we manually selected an unevenly spaced time points as the experimental observations. Data points in dynamic regions are densely sampled, while data points in steady state region are sparsely sampled. The selected time points are 0, 5, 10, 15, 20, 30, 40, 50, 75, 100, 125, 150, 175, 200, 300, 400, 600, and 800. With the measurement time points changed, we generated an experimental dataset by randomly perturbing noise-free simulation of the model, as shown in Fig. 6a where the circles represent the unevenly spaced measurement time points and the simulated experimental observations. Using this data, the iterative algorithm was performed to obtain the weights shown in Fig. 6b. In contrast to the weights in the previous examples, all data points except the first data point (*t*=0) receive very similar weights regardless of the region, dynamic or steady state. This is because the measurement time points are selected strategically make the data points roughly equally important, so that redundancy among the data points is reduced.

Using the sampling algorithm, we visualized the model predictions of acceptable parameters for these two cost functions, under unevenly spaced time points. As shown in Additional file 1: Figure S4, the belts associated to the two cost functions are quite similar to each other. This is due to the low variation among the weights shown in Fig. 6b, which makes the weighted cost function (Additional file 1: Figure S4B) and the equal-weight cost function (Additional file 1: Figure S4A) almost equivalent to each other. This example shows that the proposed weighted cost function can also be achieved by strategically selecting measurement points to avoid redundancy in the resulting experimental data.

To test the sensitivity of the iterative algorithm, we randomly simulated 100 experimental datasets, shown in Additional file 1: Figure S5A. We applied the iterative algorithm to compute weights based on each experimental dataset. Additional file 1: Figure S5B describes the variations among 100 sets of weight, where weights for the majority of the simulated datasets are close to “1” (0 in log scale), corresponding to the equal-weight cost function.

### MAPK module

To test the proposed weighted cost function in a more complex model, the MAPK module was considered [25]. This module consists of 5 variables and 9 model parameters. The ordinary differential equations of this module are depicted in Eq. (8), where *X* represents MAPK, *XE* represents the complex of *X* with the enzyme *E*, *XP* is singly phosphorylated form of MAPK, *XPE* is complex of *XP* with the enzyme, and *XPP* is doubly phosphorylated form [25]. In this example, we assume that the concentration of the enzyme *E* is initially 0.01, changes to 10 at t=1, and changes back to 0.01 at t=5. When the enzyme concentration is increased at t=1, the dynamic variables either increase or decrease, in respond to the enzyme change. To generate the experimental data, we used the model parameters in [25] as the true underlying parameters. All 9 parameters and 5 initial conditions were considered as unknown model parameters, thus the total number of parameters is 14.

8 $$ {\begin{aligned} \frac{d}{dt}[X]&=-K_{1}[X]E+ K_{2}[XE]+K_{7}[XP] \\ \frac{d}{dt}[XE]&=K_{1}[X]E-(K_{2}+k_{3})[XE] \\ \frac{d}{dt}[XP]&=K_{3}[XE]-K_{7}[XP]-K_{4}[XP]E+K_{5}[XPE]+K_{8}[XPP] \\ \frac{d}{dt}[XPE]&=K_{4}[XP]E-(K_{5}+K_{6})[XPE] \\ \frac{d}{dt}[XPP]&=K_{6}[XPE]-K_{8}[XPP] \end{aligned}}  $$

**Fig. 7:**
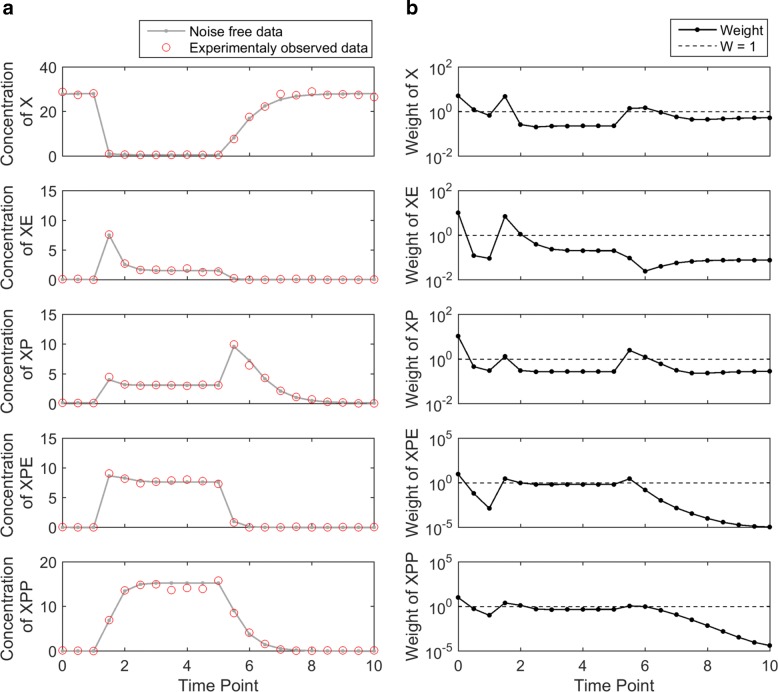
Experimental data and weights of the MAPK module. **a** The solid curve represents noise-free data obtained from the true parameter. The circles represent the noisy experimental data, generated by adding and multiplying a small amount of Gaussian noise. **b** Each dot represents the weight of a data point, and the dashed line corresponds to the equal-weight cost function. Data points in dynamically changing regions receive larger weights and the data points in flat regions receive relatively smaller weights

Figure 7a shows the simulated noise-free data generated from the true parameter and noisy experimental data obtained by randomly perturbing the noise-free data. The measurement time points are evenly spaced, every 0.5 h from 0 to 10 h. Therefore, the total number of experimental data is 105 (21 data points for each variable). When the catalyzing enzyme *E* concentration was changed at t=1 and t=5, the dynamic variables responded to the changesu. For example, the concentration of *X* decreased rapidly after t=1, while the concentrations of remaining four variables increased. As the catalyzing enzyme *E* decreased back at t=5, all variables returned to the initial condition gradually.

Using the iterative algorithm, we calculated weights for the data points, shown in Fig. 7b. Similar to the previous models, the initial data point at t=0 receives the largest weight because it is directly related to some of the unknown model parameters. The data points in the dynamic region (from t=1 to t=2) of all five variables receive the large weights. Since all five variables exhibit little dynamics from t=2 to t=5, weights of data points in these flat regions are relatively small. After t=5, weights of *X*, *XP*, *XPE*, and *XPP* slightly increased because their model predictions are changed dynamically, whereas the concentration of the *XE* barely changed and hence its data points after t=5 received small weights.

**Fig. 8:**
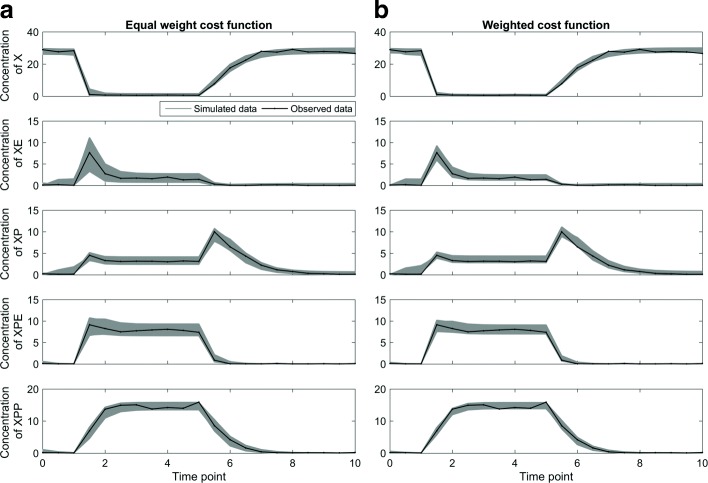
Sampling algorithm for evaluating the MAPK module. The black curves show the noisy experimental data. The gray belts show the model predictions based on the acceptable parameters obtained by the sampling algorithm. By comparing the belt width of the second variable *XE* between the two cost functions, we can see the benefit of the weighted cost function. **a** The equal-weight cost function generates imbalanced belt width between dynamic regions and flat regions. **b** The weighted cost function produces a thin belt, meaning that it is able to better constrain the model parameters to reproduce the experimental data

Figure 8 shows the results of the sampling algorithm for both cost functions. In this example, the acceptance threshold was defined as five times the cost value of the optimal parameter setting. In Fig. 8a, equal-weight cost function, the belt of variable *XE* (the second row) is quite thick in the dynamic region and thin in the flat region. This imbalanceness of acceptable model predictions between dynamic and flat regions is consistent with results in the previous examples. In Fig. 8b, the corresponding belt of variable *XE* generated with the weighted cost function is much thinner in the dynamic region, compared to the equal-weight cost function. This is because the dynamic regions receive larger weights than the flat regions. For all five dynamic variables, the belt width (variation in acceptable model predictions) from the weighted cost function is smaller than or equal to that from the equal-weight cost function.

**Fig. 9:**
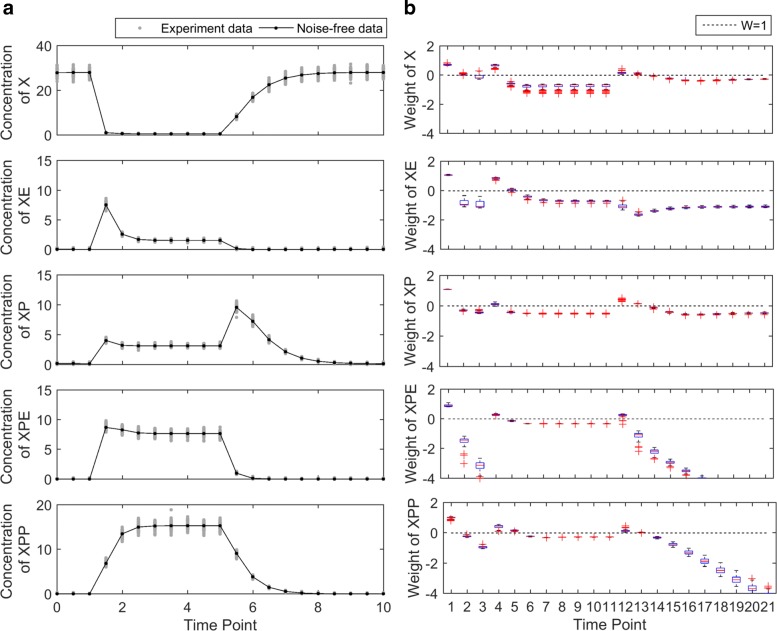
MAPK module: robustness of the uncertainty-based weights. **a** The black curve represents the noise-free data, and the gray dots represent 100 simulated noisy datasets for sensitivity analysis. **b** The dotted line indicates the equal-weight cost function. Each box shows the variation in of one weight caused by variations among the 100 noisy experimental datasets. Although some outliers exist, most weights exhibit small variations in this sensitivity analysis, showing the robustness of the uncertainty-based weights

To test the noise sensitivity of the weights in the MAPK module, we randomly generated 100 noisy time series data, as shown in Fig. 9a. We applied the iterative algorithm to compute weights starting from each of the 100 times series data. The resulting weights are shown in Fig. 9b. The first measurement time points of all variables consistently receive high weight, because they directly reveal the initial condition parameters. The variation of each weight across the 100 noisy datasets is small compared to the variation of weights across different data points, indicating robustness of the algorithm with respect to noise.

## Discussion

The motivation of our weighted cost function stems from the imbalance between high complexity of the model and limited availability of experimental data, which brings about the ill-conditioned parameter estimation. Although parameter estimation based on the proposed weighted cost function is still not perfect, the proposed weighted cost function shows its ability to reduce the redundancy in the data, leading to parameter estimation that are not skewed toward the redundant measurements in the experimental data.

In the “Results” section, two different experimental data were examined for the G1/S transition with 12 parameters: evenly spaced and unevenly spaced along the time axis. For unevenly spaced measurement data, the data at the dynamically changing region was densely sampled, and the data in the flat region was sparsely sampled. We can see that such a measurement strategy is helpful for avoiding a large amount of redundant information, which leads to ill-conditioned parameter estimation. This strategy is often adopted by biologists when designing time series experiments. Our analysis provides a mathematical perspective of why the biologists’ intuition of unevenly spaced time points is effective in time series experiments. Furthermore, by comparing the results of unevenly spaced data and evenly spaced data, we can see that strategically designing the data points to be measured can achieve equivalently effective parameter estimation, compared to the weighted cost function formulation.

## Conclusion

This paper proposed the use of weighted cost function to estimate parameters. By assigning a different weight to each data point, the relative importance of each data can be reflected when estimating the model parameters. The weight of each data point is defined by the uncertainty of each data point given the other data points. Therefore, the weight for each data point quantifies the amount of unique information it carries compared to other data points. More specifically, high weights are assigned to data points that are difficult to predict based on the other data points, whereas low weights are assigned to data points that can be accurately inferred from other data points.

To test the uncertainty-based weighted cost function, three models were examined, 6-parameter and 12-parameter G1/S transition, and MAPK module. The results show that the weighted cost function is effective in reducing the redundancy in the data, and improves parameter estimation. In order to demonstrate the benefit of the weighted cost function, we developed a sampling algorithm to efficiently identify an acceptable parameter region around the optimal parameter. This algorithm is helpful tool for sampling and sensitivity analysis.

## Additional file

Additional file 1 Supplementary Figures. This file includes all supporting figures. (PDF 434 kb)

## References

[1] Lander Arthur D (2004). A Calculus of Purpose. PLoS Biology.

[2] Sobie EA, Lee Y-S, Jenkins SL, Iyengar R (2011). Systems biology—biomedical modeling. Sci Signal.

[3] Bartocci E, Lió P (2016). Computational modeling, formal analysis, and tools for systems biology. PLoS Comput Biol.

[4] Aldridge BB, Burke JM, Lauffenburger DA, Sorger PK (2006). Physicochemical modelling of cell signalling pathways. Nat Cell Biol.

[5] Machado D, Costa RS, Rocha M, Ferreira EC, Tidor B, I R (2011). Modeling formalisms in systems biology. AMB Express.

[6] Materi W, Wishart DS (2007). Computational systems biology in drug discovery and development: methods and applications. Drug Discov Today.

[7] Fages François, Gay Steven, Soliman Sylvain (2015). Inferring reaction systems from ordinary differential equations. Theoretical Computer Science.

[8] Vilela M, Chou I-C, Vinga S, Vasconcelos ATR, Voit EO, Almeida JS (2008). Parameter optimization in s-system models. BMC Syst Biol.

[9] Anderson J, Chang T-C, Papachristodoulou A (2011). Model decomposition and reduction tools for large-scale networks in systems biology. Automatica.

[10] Meyer P, Cokelaer T, Chandran D, Kim KH, Loh PR, Tucker G, Lipson M, Berger B, Kreutz C, Raue A, Steiert B, Timmer J, Bilal E, Sauro HM, Stolovitzky G, Saez-Rodriguez J (2014). Network topology and parameter estimation: from experimental design methods to gene regulatory network kinetics using a community based approach. BMC Syst Biol.

[11] Machta BB, Chachra R, Transtrum MK, Sethna JP (2013). Parameter space compression underlies emergent theories and predictive models. Science.

[12] Deng Z, Tian T (2014). A continuous optimization approach for inferring parameters in mathematical models of regulatory networks. BMC Bioinformatics.

[13] Steiert B, Raue A, Timmer J, Kreutz C (2012). Experimental design for parameter estimation of gene regulatory networks. PLoS ONE.

[14] Transtrum MK, Qiu P (2012). Optimal experiment selection for parameter estimation in biological differential equation models. BMC Bioinformatics.

[15] Liepe J, Filippi S, Komorowski M, Stumpf MPH (2013). Maximizing the information content of experiments in systems biology. PLoS Comput Biol.

[16] Huan X, Marzouk YM (2013). Simulation-based optimal bayesian experimental design for nonlinear systems. J Comput Phys.

[17] Casey FP, Baird D, Feng Q, Gutenkunst RN, Waterfall JJ, Myers CR, Brown KS, Cerione RA, Sethna JP (2007). Optimal experimental design in an epidermal growth factor receptor signalling and down-regulation model. IET Syst Biol.

[18] Busetto AG, Hauser A, Krummenacher G, Sunnaker M, Dimopoulos S, Ong CS, Stelling J, Buhmann JM (2013). Near-optimal experimental design for model selection in systems biology. Bioinformatics.

[19] Vanlier J, Tiemann CA, Hilbers PAJ, van Riel NAW (2012). A bayesian approach to targeted experiment design. Bioinformatics.

[20] Vanlier J, Tiemann CA, Hilbers PAJ, van Riel NAW (2012). An integrated strategy for prediction uncertainty analysis. Bioinformatics.

[21] Pauwels E, Lajaunie C, Vert J-P (2014). A bayesian active learning strategy for sequential experimental design in systems biology. BMC Syst Biol.

[22] Potra FA, Wright SJ (2000). Interior-point methods. J Comput Appl Math.

[23] van Ravenzwaaij D, Cassey P, Brown SD (2016). A simple introduction to markov chain monte–carlo sampling. Psychon Bull Rev.

[24] Swat M, Kel A, Herzel H (2004). Bifurcation analysis of the regulatory modules of the mammalian g1/s transition. Bioinformatics.

[25] Voit E (2012). A First Course in Systems Biology, 1st ed.

