# Supplementary material for: Quantifying the relative importance of experimental data points in parameter estimation
Source: BMC Syst Biol. 2018 Nov 22;12(Suppl 6):103. doi: 10.1186/s12918-018-0622-6 (PMC6249737; doi:10.1186/s12918-018-0622-6)
Supplement: Supplementary file 1 — Supplementary Figures. This file includes all supporting figures. (PDF 434 kb) [file 12918_2018_622_MOESM1_ESM.pdf]

Supplementary Figures

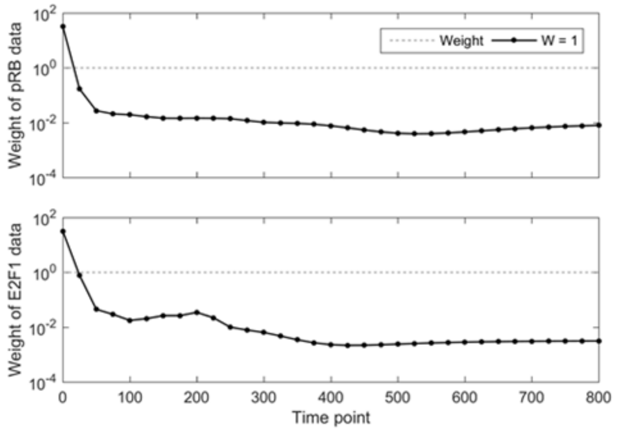

Figure S1 Weights of the G1/S transition model with 12-parameters. Each dot represents the weight of a data point, and the dashed line corresponds to the weights in the equal-weight cost function.

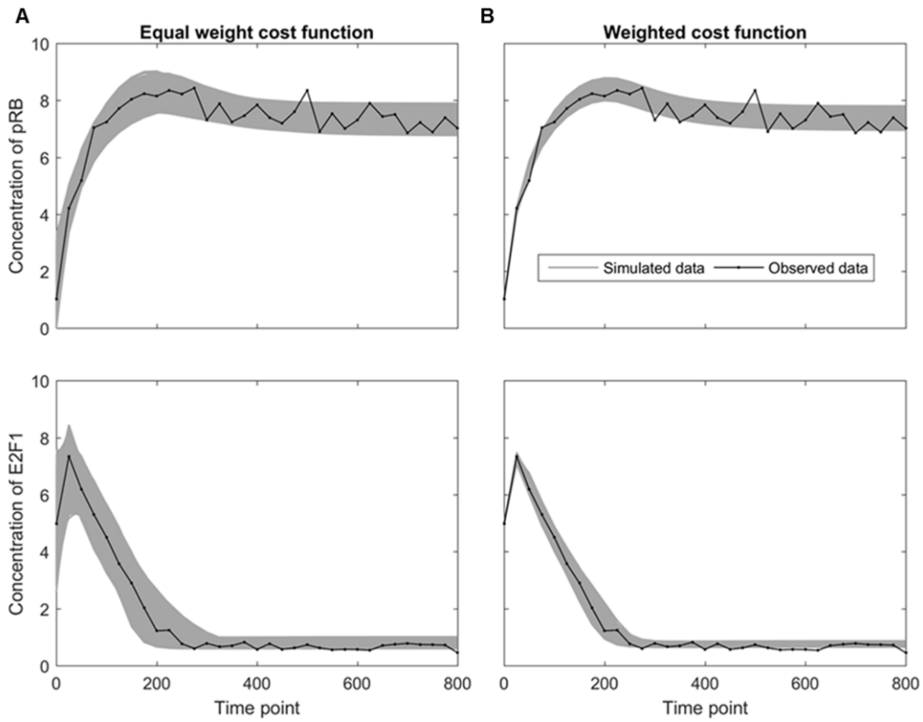

Figure S2 Sampling algorithm for evaluating G1/S transition with 12-parameters. The black curve represents experimental data and gray curves represent simulated datasets obtained from the acceptable parameters. The collections of gray curves form the gray belts, each representing the variation of model prediction of one variable, based on the acceptable parameters obtained by the equal-weight or weighted cost function. A) The equal-weight cost function leads to highly unbalanced belt width because of the large number of data points in the steady state region. B) The weighted cost function leads to thinner belts, indicating that the weighted formulation is able to better constrain the estimated parameters.

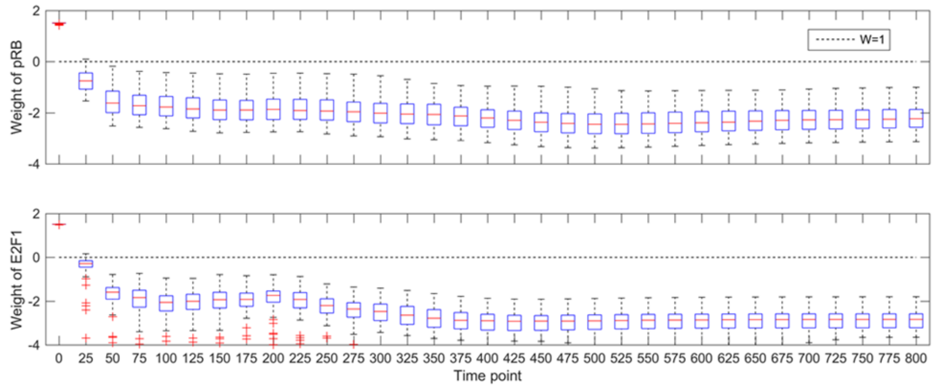

Figure S3 G1/S transition with 12-parameters: robustness of the uncertainty-based weights. The dotted line represents the equal-weight cost function. Each box represents the weights for one data point, computed from the 100 noisy experimental datasets.

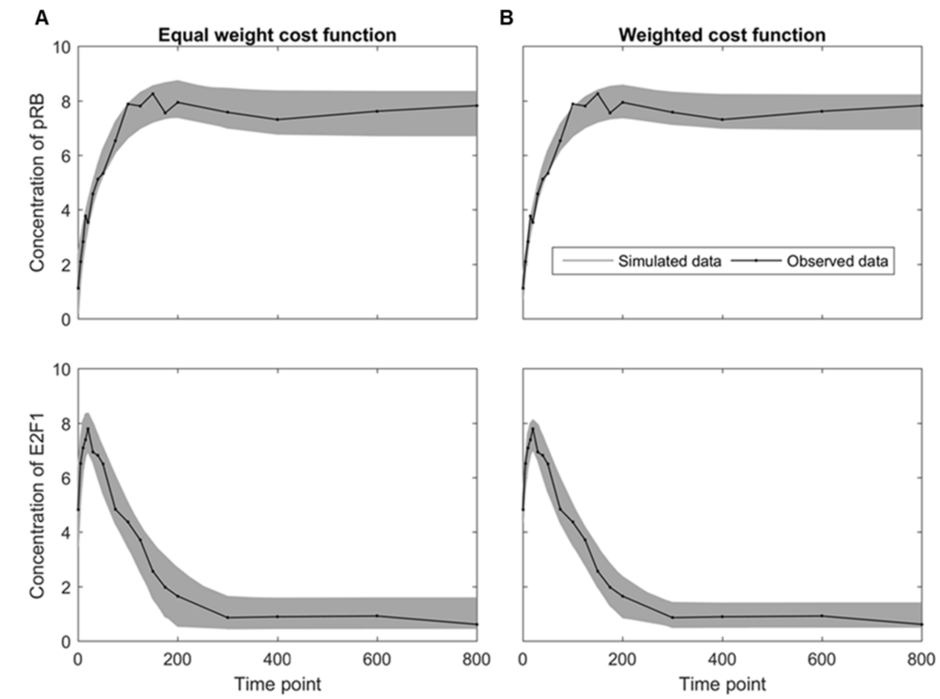

Figure S4 Sampleing algorithm for evaluating G1/S transition with unevenly spaced time points. A) Equal-weight cost function. B) Weighted cost function. The black curve shows the noisy experimental data and the gray belts show the model predictions based on the acceptable parameter setting obtained by the sampling algorithm. Both cost functions lead to similar belt, indicating that the strategically chosen time points reduce the redundancy in the data, similar to the weighted cost function based on uncertainty.

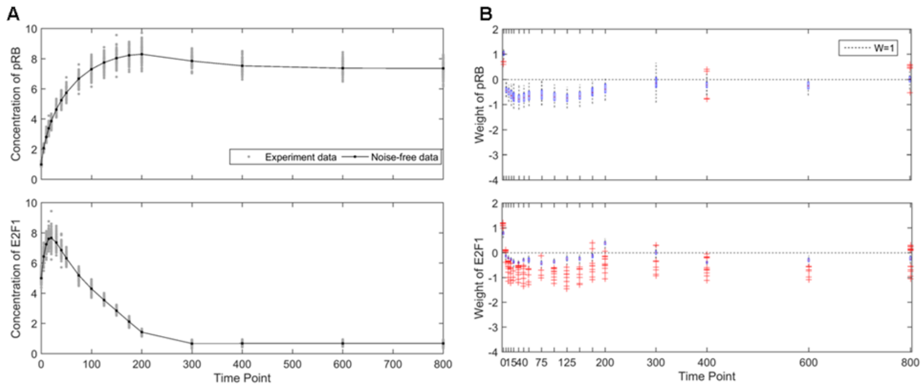

Figure S5 G1/S transition with unevenly spaced time points: robustness of the uncertainty-based weights. A) The black curve represents the noise-free data and the grey dots represent 100 simulated noisy datasets for sensitivity analysis. B) The dotted horizontal line represents the equal-weight cost function, and each box shows the weights for one data point computed from the 100 noisy experimental datasets. Although some outliers exist, majority of the weights are very close to the dotted line, meaning that the weights are robust to the simulation noise.
